# Immunomodulatory effects of testosterone and letrozole during *Plasmodium berghei* ANKA infection

**DOI:** 10.3389/fcimb.2023.1146356

**Published:** 2023-06-13

**Authors:** Teresita de Jesús Nolasco-Pérez, Luis Antonio Cervantes-Candelas, Fidel Orlando Buendía-González, Jesús Aguilar-Castro, Omar Fernández-Rivera, Víctor Hugo Salazar-Castañón, Martha Legorreta-Herrera

**Affiliations:** ^1^ Laboratorio de Inmunología Molecular, Unidad de Investigación Química Computacional, Síntesis y Farmacología en Moléculas de Interés Biológico, División de Estudios de Posgrado e Investigación, Facultad de Estudios Superiores Zaragoza, Universidad Nacional Autónoma de México (UNAM), Ciudad de México, Mexico; ^2^ Posgrado en Ciencias Biológicas, Universidad Nacional Autónoma de México, Ciudad de México, Mexico

**Keywords:** *Plasmodium berghei* ANKA, malaria, sex hormones, androgen, testosterone, letrozole, immune response, parasite

## Abstract

**Introduction:**

Malaria is one of the leading health problems globally. Plasmodium infection causes pronounced sexual dimorphism, and the lethality and severity are more remarkable in males than in females. To study the role of testosterone in the susceptibility and mortality of males in malaria, it is common to increase its concentration. However, this strategy does not consider the enzyme CYP19A1 aromatase, which can transform it into oestrogens.

**Methods:**

To avoid the interference of oestrogens, we inhibited in vivo CYP19A1 aromatase with letrozole and increased the testosterone level by exogen administration before infection with Plasmodium berghei ANKA. We measured the impact on free testosterone, 17β-oestradiol and dehydroepiandrosterone levels in plasma; additionally, we evaluated parasitaemia, body temperature, body mass, glucose levels and haemoglobin concentration. Furthermore, we evaluated the effects of testosterone on the immune response; we quantified the CD3+/CD4+, CD3+/CD8+, CD19+, Mac-3+ and NK cells in the spleen and the plasma concentrations of the cytokines IL-2, IL-4, IL-6, IFN-, IL-10, TNF-α and IL-17A. Finally, we quantified the levels of antibodies.

**Results:**

We found that mice treated with the combination of letrozole and testosterone and infected with Plasmodium berghei ANKA had increased concentrations of free testosterone and DHEA but decreased levels of 17β-oestradiol. As a result, parasitaemia increased, leading to severe anaemia. Interestingly, testosterone increased temperature and decreased glucose concentration as a possible testosterone-mediated regulatory mechanism. The severity of symptomatology was related to critical immunomodulatory effects generated by free testosterone; it selectively increased CD3+CD8+ T and CD19+ cells but decreased Mac-3+. Remarkably, it reduced IL-17A concentration and increased IL-4 and TNF-α. Finally, it increased IgG1 levels and the IgG1/IgG2a ratio. In conclusion, free testosterone plays an essential role in pathogenesis in male mice by increasing CD8+ and decreasing Mac3+ cells and mainly reducing IL-17A levels, which is critical in the development of anaemia. Our results are important for understanding the mechanisms that regulate the exacerbated inflammatory response in infectious diseases and would be useful for the future development of alternative therapies to reduce the mortality generated by inflammatory processes.

## 1 Introduction

*Plasmodium* is the causative agent of malaria, the deadliest global parasitic disease, producing more than 241 million new clinical cases and 627,000 deaths in 2020 alone (WHO, 2021). The severity of clinical manifestations and mortality is almost six times higher in male than in female (Benten et al., 1992; Zhang et al., 2000; Cernetich et al., 2006). This demonstrates the marked sexual dimorphism in malaria and suggests the involvement of estrogens and androgens in this phenomenon. The most important estrogen in female patients is 17β-estradiol, while testosterone is the most relevant and concentrated androgen in male patients. Both hormones have immunomodulatory properties and are synthesized from cholesterol in both sexes (Sanderson, 2006). Nevertheless, estradiol has immunostimulatory properties, while testosterone has immunosuppressive activity (Wichmann et al., 1996). Gonadectomy that reduces the concentration of both sex hormones by eliminating their primary site of synthesis increases parasitemia in female mice infected with *Plasmodium berghei* ANKA (*Pb* ANKA), while the opposite occurs in male patients. Furthermore, gonadectomy increases the macrophage population and decreases NK cells only in *Pb* ANKA-infected male mice (Legorreta-Herrera et al., 2015). This suggests that both cell populations are under androgen regulation, but female and male patients are affected differently. Recently, the effects of administering 17β-estradiol to intact or gonadectomized mice have been documented; increasing the concentration of this steroid raises parasitemia in both sexes but increases the number of CD8^+^ and NK cells only in female mice (Cervantes-Candelas et al., 2021b). In addition, blocking the estrogen receptor *in vivo* in female mice increases parasitemia, aggravates pathology, augments CD8^+^ and B220^+^ populations and decreases IL-2, IL-6, and IL-17 concentrations (Cervantes-Candelas et al., 2021a). Relative to testosterone, it has been described that it exerts its effects by binding to the androgen receptor (AR), which functions as a transcription factor and alters the expression of different genes (genomic mechanism) (Brinkmann, 2011). In addition, androgens cause other nongenomic effects faster through aromatization to estrogens (Rahman and Christian, 2007). In contrast, androgen ablation increases the synthesis of proinflammatory cytokines in dendritic cells (Sellau et al., 2019). Furthermore, testosterone suppresses the immune response (Wunderlich et al., 2002) and reduces TNF-α synthesis in a dose-dependent manner (Chen et al., 2016). The testosterone concentration is a determining factor in the regulation of inflammatory processes that affect cell proliferation and differentiation and suppress cytokine synthesis (Bianchi, 2019).

Regarding the effects of testosterone on the immune response in malaria, it has been documented that increasing its concentration in mice infected with *Plasmodium chabaudi* (nonlethal strain) limits the mechanisms that eliminate the parasite, resulting in the death of mice (Wunderlich et al., 1991). This increased susceptibility to *P. chabaudi* persisted even 12 weeks after hormone administration was halted, and the outcome was associated with decreased antibody concentration; however, no changes in cytokine synthesis were detected (Benten et al., 1997). Interestingly, this effect does not disappear in the presence of androgen receptor blockers, suggesting that the effects of the hormone do not always depend on the interaction with its receptor (Benten et al., 1992). This left many questions about the mechanisms involved in testosterone immunosuppression in malaria. Recently, it was reported that gonadectomy increases parasitemia in *Pb* ANKA-infected CBA/Ca mice of both sexes, and reconstituting gonadectomized mice with testosterone prevents weight loss and hypothermia caused by infection with the parasite, but affects CD4^+^, CD8^+^, and B220^+^ cells differently depending on sex and testosterone concentration (Aguilar-Castro et al., 2022). Although the experiments described above describe the effects of testosterone on the pathology and/or immune response in murine malaria, the authors did not consider that administering testosterone increases the concentration of the aromatase substrate, the enzyme that converts testosterone to estradiol, and thus also increases estrogen concentration, making it difficult to interpret the effects produced by testosterone and those that are a consequence of increasing estrogen concentration. In this work, we inhibited aromatase *in vivo* with letrozole, a highly selective inhibitor of this enzyme (Bulun et al., 2003; Geisler and Lonning, 2005), and measured the effects of letrozole and testosterone on pathology and the immune response against *Pb* ANKA. We first assessed the concentrations of free testosterone, 17β-estradiol, and dehydroepiandrosterone (DHEA). In addition, we evaluated the effects of letrozole and testosterone on disease severity by measuring body temperature, weight loss, glucose levels, and hemoglobin concentration (a measure of anemia). Regarding the immune response, we quantified CD3^+^, CD3^+^CD4^+^, CD3^+^CD8^+^, CD19^+^, macrophages, and NK cell populations in the spleen and the concentrations of pro- and anti-inflammatory cytokines in the spleen. Finally, we also measured the concentrations of IgM, IgG1, IgG2a, IgG2b, and IgG3 antibodies in the serum of mice. These results shed light on the involvement of testosterone in the immune response against parasitic diseases and should be considered in the design of distinct antimalarial treatments for male and female patients.

## 2 Materials and methods

### 2.1 Mice

CBA/Ca mice were a generous gift from Dr. William Jarra (National Institute for Medical Research, London, UK). Mice were kept, fed, raised, and maintained in an environment free of specific pathogens at the animal house facilities from Facultad de Estudios Superiores Zaragoza, Universidad Nacional Autónoma de México (UNAM), Mexico. The protocol received approbation by the Local Ethics Committee (registration number 28/04/SO/3.4.1) according to the Mexican official standard NOM-94 062-ZOO-1999 for the use and care of laboratory animals.

### 2.2 Letrozole and testosterone administration

Letrozole (Sigma-Aldrich, St. Louis, MO, USA) was pulverized, and a suspension was prepared with almond oil (Zential, Italy). Each mouse was subcutaneously injected with a previously calibrated dose of letrozole (7 mg/kg body weight) in a total volume of 30 µl daily for 14 days preinfection, on the day of infection, and 6 days postinfection, for a total of 21 administrations. To determine the dose of letrozole used in this work, a dose–response curve was generated with the following doses: 0, 1, 2, 4, and 7 mg/kg (**Supplementary Figure S1**). In addition, the potency (increase in free testosterone concentration) of the administration of letrozole, testosterone, and the combination of both compounds was evaluated, which corroborates that the doses used in the combination synergistically increase the potency of letrozole or testosterone compared to when administered separately (**Supplementary Figures S2**, **S3**).

Each mouse was administered 30 mg/kg testosterone (Schering Plough, Newton, NJ, USA) or vehicle [sweet almond oil (Zential, Italy)] subcutaneously every 72 h for 3 weeks as previously was published (Benten et al., 1991; Benten et al., 1992; Aguilar-Castro et al., 2022). The day after the last testosterone treatment, mice were infected with *Pb* ANKA. In addition, groups treated with the combination of letrozole and testosterone (letrozole + testosterone) received 7 mg/kg letrozole and 30 mg/kg testosterone in 30 μl of vehicle subcutaneously as described above.

### 2.3 Parasite and infection

*Plasmodium berghei* ANKA was a generous gift from Dr. William Jarra (National Institute of Medical Research, Mill Hill, London, UK). The parasite was cryopreserved in liquid nitrogen until use. To infect the mice, a vial of parasite was first thawed, and the content was immediately injected intraperitoneally into a 4-week-old mouse to activate the parasite. When parasitemia reached approximately 20%, a blood sample was taken, and the number of total erythrocytes was quantified in a Neubauer chamber. To assess parasitemia, a drop of blood was spread on a slide, fixed with absolute methanol (Sigma-Aldrich) and stained with Giemsa (Merck, Darmstadt, Germany). To determine the percentage of parasitemia, a total of 200 erythrocytes were counted in triplicate. Using these data, an inoculum containing 1×10^3^ parasitized erythrocytes in 100 µl of PBS was administered into the caudal vein of the tail of each mouse. This ensured that all parasites reached the bloodstream at the same time.

### 2.4 Quantification of testosterone, 17β-estradiol, and DHEA

On the day of sacrifice, mouse blood was recovered in heparinized tubes. The blood was centrifuged at 1,000 × *g* for 5 min; the plasma was separated and frozen at −20°C until use. One hundred microliters of plasma was mixed with 5 ml of ethyl ether (JT Baker, Fisher Scientific SL, USA) and shaken vigorously for 5 min. The aqueous phase was frozen on dry ice mixed with ethanol (Sigma-Aldrich). The organic phase was transferred to a glass tube, and ether was evaporated in a water bath for 48 h. The extract was rehydrated with 1,000 ml of ethanol (Sigma-Aldrich). The steroidal extract was rehydrated with 1,000 µl of PBS/0.1% gelatine (Sigma-Aldrich).

Free testosterone was assessed using the EIA-2924 kit with a sensitivity of 0.04 pg (DRG International, Frauenbergstr, Germany) following the commercial protocol. Testosterone levels were calculated using a standard curve included in the kit. Briefly, 20 µl of the steroidal extract or standard curve sample and 100 μl of the conjugate were added to each plate well and incubated for 1 h at 37°C. The plate was carefully rinsed with a washing solution. Then, 100 µl of tetramethylbenzidine (TMB) was added and incubated at 28°C in the dark for 15 min. Next, 100 μl of stop solution was added, and after 5 min, the plate absorbance was read at 620 nm on a Multiskan Ascent 96 plate reader (Thermo Fisher Scientific).

17β-estradiol was quantified with a Siemens Immulite LKE2 solid-phase competitive chemiluminescent immunoassay (detection range: 20–2,000 pg/ml, sensitivity: 15 pg) (Immulite 1000 Siemens Llanberis Gwynedd, UK). Five hundred microliters of the sample extract was added to the reaction cuvettes and processed by automated equipment. The results were measured on the Immulite 1000 Immunoassay System (Siemens Healthineers, Surrey, UK).

Dehydroepiandrosterone (DHEA) was quantified using DHEA EIA-3415 (detection range: 0.07–30 ng/ml, sensitivity: 0.07 ng) (DRG International). Ten microliters of extract or 10 µl of each standard with 100 μl of conjugate per well was added to the plate. It was incubated for 1 h at room temperature. Subsequently, the plate was washed four times with 400 μl of wash solution. TMB (100 µl) was added as a substrate and incubated at 28°C for 15 min. The reaction was halted with 100 μl of stop solution, and after 5 min, the absorbance was read at 630 nm using a Multiskan Ascent 96 plate reader (Thermo Fisher Scientific). DHEA levels were calculated using a standard curve included in the kit.

### 2.5 Parasitemia

Blood smears were prepared daily, fixed with absolute methanol (Sigma-Aldrich), and stained with Giemsa (Merck). Parasitemia assessment was performed using the oil 100× objective of a Zeiss Standard 20 microscope (Carl Zeiss LTD, Welwyn Garden City, UK). When the number of parasitized red blood cells reached 0.5%, 200 red blood cells were counted. Lower levels of parasitemia were estimated by considering the parasitized erythrocytes present in 50 fields. The course of infection in each group is displayed as the geometric mean of the parasitemia in each group.

### 2.6 Severity of disease

The severity of disease was assessed by measuring body temperature, body mass loss (cachexia), glucose concentration, hemoglobin levels (as an indicator of anemia), and parasitemia. Following *Pb* ANKA infection, mice were monitored daily to document mortality.

#### 2.6.1 Change in body temperature

Body temperature was evaluated on day 0 to day 8 after infection at the same time utilizing an infrared thermometer (Thermofocus, 01500A/H1N1, Vedano Olana-Varese, Italy). The thermometer beam was directed at 5 cm from the intraperitoneal region of each mouse. Every bar represents the mean ± SEM in every single group.

The change in body temperature was calculated using the following equation:


$$Change\;\left(percentage\right)=\;\frac{\left(Vx-V0\right)*100}{V0}$$

were *Vx* is the body temperature in the mouse ion that day and *V*0 is the temperature on the day of infection.

#### 2.6.2 Body mass loss

Mice were weighed daily at the same time of day using an electronic scale (Ohaus, Parsippany, NJ, USA). The body weight on the day of infection (day zero) was 100%. The change in body mass was calculated as the weight of the mouse on each day compared to the weight on day zero, such as body temperature.

#### 2.6.3 Glucose quantification

A drop of blood from the tail of each mouse was applied daily to the glucometer test strip (ACCU-CHEK, Performa, Roche ^®^), the glucose concentration was recorded on the display, and the detection range of the system was 10–600 mg/dl.

#### 2.6.4 Quantification of hemoglobin levels

The hemoglobin (Hb) concentration was measured by a colorimetric method. Briefly, 2 µl of blood was added to 498 µl of Drabkin’s reagent (Sigma-Aldrich). The samples were mixed and incubated for 5 min in the dark. Finally, the absorbance at 540 nm was measured in a plate reader (Multiskan GO, Thermo Fisher Scientific, Inc. Waltman, MA, USA). The Hb concentration was calculated using a commercial hemoglobin standard.

### 2.7 Splenic index

On day 8 after *Pb* ANKA infection, mice were weighed on an electronic balance (Ohaus) and sacrificed, and the spleen was removed and weighed on an analytical balance (Sartorius, Göttingen, Germany). To calculate the splenic index, the spleen weight value was divided by the weight of the mouse, and the data are presented as the mean ± SEM.

### 2.8 Quantification of spleen cell populations using flow cytometry

To quantify the cell subpopulations in the spleen, we used multicolor flow cytometry as previously described (Aguilar-Castro et al., 2022). Briefly, mice were sacrificed 8 days postinfection, their spleens were removed, and cells were disaggregated with a nylon net. The spleen cells were washed with cold PBS and fixed with a commercial solution (Becton and Dickinson, Franklin Lakes, NJ, USA). The cells were washed with PBS, 1% albumin, and 0.1% NaN_3_ and counted in a Neubauer chamber. A total of 1×10^6^ cells were stained in the dark for 30 min using the following previously calibrated anti-mouse fluorochrome-coupled antibodies: FITC-antiCD3, APC-antiCD4, PE-antiCD8, APC-anti CD19, PE-antiMac3, and CD16^+^/32^+^. For staining, three mixtures of antibodies were set up: the first identified T cells (CD3^+^, CD4^+^, and CD8^+^) and the second mixture identified B cells and macrophages (CD19^+^ and Mac3^+^). The third mixture (CD19^-^CD3^-^CD16^+^/32^+^) was used to identify NK cells. We acquired 10,000 events per sample, then plotted FSC vs. SSC and selected the region corresponding to lymphocytes to eliminate doublets and singlets; this first gate was considered 100%. We selected FITC-CD3^+^ T cells from this region by plotting SSC vs. FITC-CD3^+^ and calculated the relative percentage to the first gate to exclude B^+^ and NK^+^ cells. Two additional dot plots were created from the first gate, corresponding to CD4^+^ and CD8^+^ T cells (SSC vs. APC-CD4^+^ and SSC vs. PE-CD8^+^ respectively), and the proportions were calculated relative to the initial lymphocyte gate. The second mixture was prepared to identify B cells and macrophages using APC-antiCD19 and PE-antiMac3 antibodies, respectively. The CD19^+^ population was identified from the lymphocyte region utilizing an FSC vs. SSC dot plot that corresponded to 100% and an SSC vs. APC-CD19 dot plot. Monocytes were identified by plotting FSC and SSC corresponding to a population over lymphocytes, and this region was 100%, excluding CD3^+^, CD19^+^, and NK populations. Mac3^+^ cells were identified from this region with a dot blot of SSC vs. PE-antiMac3.

The third mixture was prepared to identify NK cells, and we used four dot blots. In the first dot blot, NK and CD3^+^ cells were selected by potting SSC vs. FSC; the second dot blot used the FITC-antiCD3 antibody to select CD3^-^ cells; and in the third dot blot, APC-antiCD19 antibody was utilized to select CD19^-^ cells. In the fourth dot blot, NK cells were selected by plotting SSC against PE-CD16^+^/32^+^. All antibodies were acquired from BioLegend (San Diego, CA, USA). The cells were washed with PBS and resuspended in 100 μl of FACS solution. Stained cells were analyzed using the flow cytometer FACS Aria II (BD Biosciences, San Jose, California, USA). Data were processed with FlowJo™ software (Beckton and Dickinson, Ashland, OR, USA).

### 2.9 Th1/Th2/Th17 cytokine quantification

On day 8 postinfection, mice were sacrificed, and heart blood was recovered into heparinized tubes and centrifuged at 2,000 × *g* for 15 min. Plasma was recuperated and frozen at −70°C until use. The levels of IFN-γ, TNF-α, IL-2, IL-4, IL-5, IL-6, IL-10, and IL-17A were assessed using the cytometric bead array (BD mouse Th1/Th2/Th17 cytokine CBA Kit Biosciences-Pharmingen, Heidelberg, Germany) according to the manufacturer’s procedure, except that the assay was performed in microtubes. The standard curve began at 0.625 pg/ml. The sensitivity achieved with this modification was 0.9 ± 0.05 pg/ml, and the interassay variation was 5%.

### 2.10 Antibody-level measurement

We used a previously described method (Legorreta-Herrera et al., 2004). Briefly, 1 µg of *Pb* ANKA antigen diluted in 100 µl of carbonate buffer was added to each well of the ELISA microplate (Corning, NY, USA) and incubated for 2 h at 37°C. The plate was washed with 0.5% Tween 20 in PBS and blocked with 3% skimmed milk in PBS for 2 h at 37°C. The plate was washed and incubated with 100 µl of (1:20) diluted test plasma for 1 h at 37°C; the plate was washed and precalibrated dilutions of Ab goat anti-mouse specific for IgM, IgG, IgG1, IgG2a, IgG2b, and IgG3 (Zymed, San Francisco California, USA) were added for 1 h at 37°C; the plates were washed and incubated with streptavidin-peroxidase (Sigma-Aldrich) after washing; and the plates were incubated with ortho-phenylenediamine at 0.4 mg/ml in citrate buffer with 0.03% hydrogen peroxide and incubated for 20 min in the dark. The absorbance was measured at 492 nm using a Stat-Fax 2100 microplate reader (Awareness Technology Inc. USA).

### 2.11 Experimental design

To analyze the immunomodulatory effect of testosterone in malaria, we administered the hormone to male mice; nevertheless, as this strategy could promote testosterone conversion to estrogens by the enzyme P450 aromatase (aromatase), we inhibited the enzyme *in vivo* with letrozole and analyzed its effects on the immune response in an experimental malaria model. Three-month-old male CBA/Ca mice were organized into five groups. The first group was untreated, the second was administered almond oil as a vehicle (vehicle), the third group received letrozole at the previously calibrated dose (7 mg/kg body weight) for 3 weeks, the fourth group was treated with testosterone (30 mg/kg body weight), and the fifth group received the combination of letrozole and testosterone at the same doses.

Mice in each group were separated into two subgroups; only one subgroup was infected with 1×10^3^ erythrocytes parasitized with *Pb* ANKA. All mice were sacrificed 8 days later.

### 2.12 Statistical analysis

The significant difference between groups was determined by one-way analysis of variance (ANOVA), except for the percentage parasitemia data, which were calculated by repeated means analysis. We used the Bonferroni *post-hoc* test on all results. A significant difference between groups was considered at *p* ≤ 0.05 (*n* = 5). The area under the curve (AUC) analysis represented the total area obtained per group of the corresponding determination from day 0 to day 8 postinfection. All analyses were performed in GraphPad Prism version 9.5.1 (Graph Pad Software, San Diego, CA, USA). The whole experiment was conducted twice.

## 3 Results

### 3.1 Testosterone and letrozole administration increased free testosterone and DHEA levels

**Figure 1:**
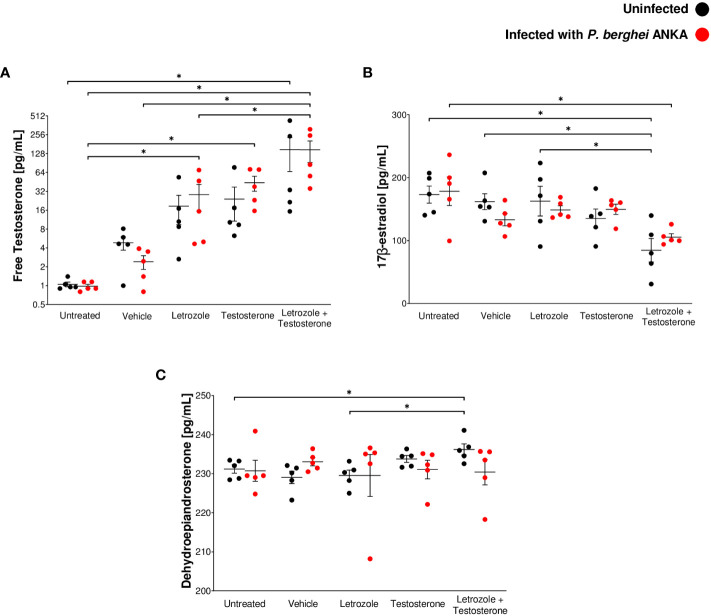
The combination of letrozole and testosterone increased the concentration of free testosterone and DHEA but decreased 17β-estradiol levels. Ten groups of 10 CBA/Ca male mice were treated with letrozole, testosterone, or the combination of letrozole and testosterone (letrozole + testosterone) for 3 weeks, and two additional groups (untreated and vehicle-treated) were used as controls. Mice in each group were separated into two subgroups (*n* = 5); only one subgroup in each group was infected with *Pb* ANKA. Mice were sacrificed at day 8 postinfection and plasma was used to quantify steroid levels. **(A)** Levels of free testosterone. **(B)** 17β-estradiol levels and **(C)** DHEA levels. Each point shows the individual result of each mouse ± SEM (*n* = 5). Asterisks (*) represent significant differences between the two groups at *p* ≤ 0.05. Statistical analysis of one-way ANOVA and Bonferroni *post-hoc* test. The whole experiment was repeated twice.

The immunomodulatory activity of testosterone in malaria is concentration dependent (Aguilar-Castro et al., 2022). Nevertheless, when testosterone is administered exogenously, it is possible to increase the activity of the aromatase enzyme, which, in turn, would increase the levels of 17β-estradiol that would mask testosterone activity. Therefore, in this study, we inhibited aromatase *in vivo* using letrozole and assessed the impact on the levels of free testosterone, 17β-estradiol, and dehydroepiandrosterone (DHEA), a testosterone precursor hormone, on day 8 postinfection. Infection did not modify the concentration of the three hormones (**Figure 1**). Administering testosterone or letrozole increased the free testosterone concentration compared to the untreated infected group. Groups receiving letrozole + testosterone had a twofold increase in free testosterone concentration compared to groups receiving letrozole or testosterone separately (**Figure 1A**).

Regarding 17β-estradiol and DHEA levels, administration of letrozole or testosterone alone to *Pb* ANKA-infected mice did not modify the levels of both hormones compared with the vehicle-treated infected group (**Figures 1B, C**). In addition, infected mice administered the combination of letrozole + testosterone exhibited decreased levels of 17β-estradiol (**Figure 1B**). Finally, mice treated with letrozole + testosterone uninfected exhibited higher levels of DHEA than untreated and letrozole-treated mice in the same condition (**Figure 1C**). These data show that the administration of letrozole + testosterone to *Pb* ANKA-infected mice generated a dramatic increase in free testosterone levels and a significant decrease in 17β-estradiol.

### 3.2 Parasitemia of CBA/Ca mice infected with *Pb* ANKA increases directly in proportion to levels of free testosterone

**Figure 2:**
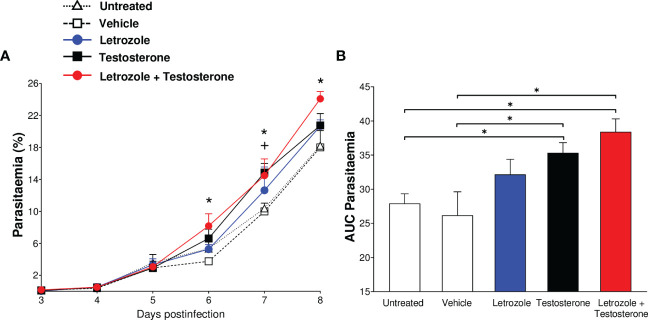
The combination of letrozole + testosterone increases the parasitemia of *Plasmodium berghei* ANKA. Five groups of CBA/Ca male mice were treated with letrozole, testosterone, or a combination of letrozole and testosterone. Two groups (untreated and vehicle-treated) were used as controls. All mice were infected with *Plasmodium berghei* ANKA, and the day of infection was considered day 0. Mice were sacrificed on day 8 postinfection. Daily, from day 3 to day 8, parasitemia was assessed. **(A)** Each dot represents the geometric mean of the percentage of parasitized erythrocytes ± SEM of each group. **(B)** Each bar represents the area under the curve (AUC) from day 3 to day 8 postinfection of each group ± SEM. The asterisk (*) represents a statistically significant difference between the letrozole and testosterone groups and the control groups. The cross (+) represents the significant difference between the group treated with testosterone and the control groups with *p* ≤ 0.05 (*n* = 5). To calculate the significant differences between groups, repeated means analysis was used in **(A)** while in **(B)**, we use one-way ANOVA. For both graphs, we used the Bonferroni *post-hoc* test. The whole experiment was repeated twice.

To confirm the impact of testosterone levels on parasitemia, CBA/Ca mice given letrozole, testosterone, or letrozole + testosterone were infected with *Pb* ANKA and groups of untreated or vehicle-treated infected mice were used as controls. The groups treated with testosterone or with letrozole + testosterone groups showed increased parasitemia on days 6, 7, and 8 postinfection compared to the untreated infected group; additionally, infected mice treated only with testosterone showed increased parasitemia on day 7 postinfection compared to the untreated infected group (**Figure 2A**). To analyze the overall result of parasitemia on all days of infection, the area under the curve (AUC) for each group was calculated and plotted as a histogram. This analysis revealed that mice treated with testosterone or with testosterone + letrozole had increased parasitemia compared to the vehicle-treated infected group. This finding confirms that increasing testosterone concentration enhances parasitemia in male mice infected with *Pb* ANKA (**Figures 1A**, **2B**).

### 3.3 Testosterone increases body temperature and body mass but reduces glucose and hemoglobin levels in mice infected with *Pb* ANKA

To analyze the effects of testosterone on the severity of infection with *Pb* ANKA, we assessed daily the body temperature to detect hypothermia, the body mass change to examine cachexia, the levels of glucose to examine hyperglycemia, and hemoglobin concentration to examine anemia, all parameters related to the pathology and severity of *Plasmodium* infection (Lamikanra et al., 2007; Thawani et al., 2014; Adedemy et al., 2015). In addition, to evaluate the overall effect of each variable, we calculated and plotted the AUC of all variables.

**Figure 3:**
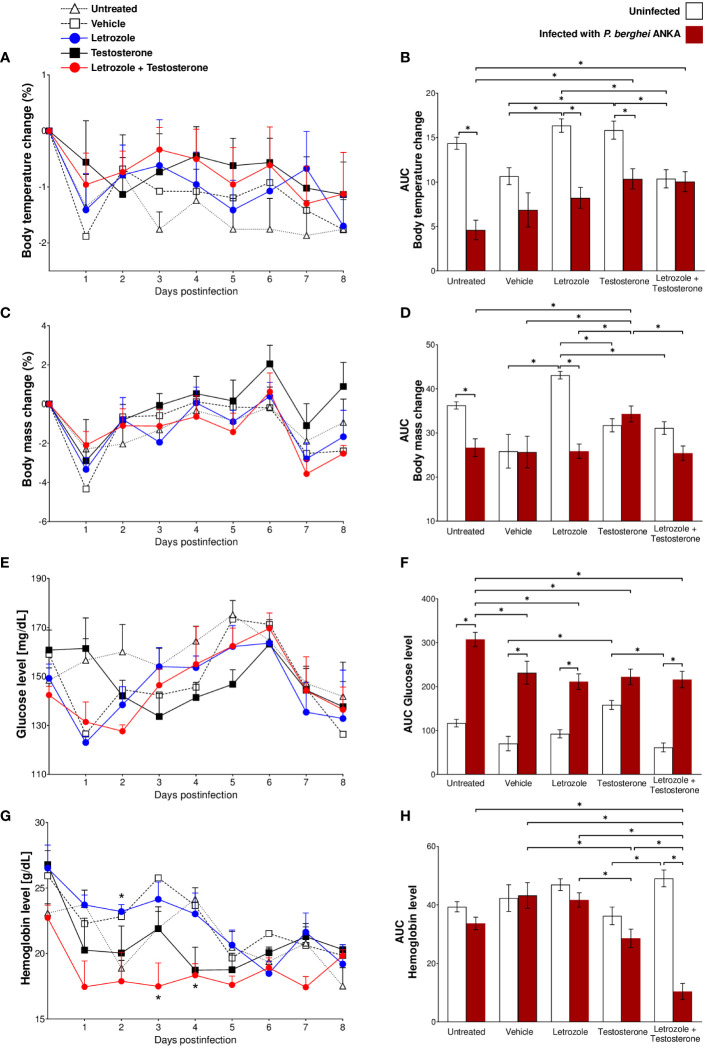
Letrozole and testosterone administration regulated body temperature and body mass and decreased hemoglobin and glucose concentrations in CBA/Ca mice infected with *Pb* ANKA. Five groups of male CBA/Ca mice (*n* = 10) were given letrozole, testosterone, or a combination of letrozole and testosterone. Untreated and vehicle-treated groups were used as controls. Half of the total number of mice in all groups were infected with *Pb* ANKA, and the day of infection was considered day 0 postinfection. All mice were sacrificed at day 8 PI. Daily from day 0 to day 8 postinfection, the mass and body temperature of all mice were recorded, and the change in mass and body temperature was calculated based on day 0 postinfection. The line graphs represent the average value of infected groups ± SEM from day 0 to day 8 postinfection. Each bar represents the area under the curve (AUC) calculated from day 0 to day 8 PI of each group ± SEM (*n* = 5). **(A, B)** Body temperature change; **(C, D)** body mass change; **(E, F)** glucose levels; **(G, H)** hemoglobin levels. Asterisks (*) represent significant differences between the two groups at *p* ≤ 0.05. Line graphs were analyzed using repeated means with Bonferroni *post-hoc* test, and the bar charts were analyzed using one-way ANOVA with Bonferroni *post-hoc* test. The whole experiment was repeated twice.

Infection reduced the body temperature in the untreated and vehicle-, letrozole-, and testosterone-treated groups. Interestingly, the infected group administered letrozole + testosterone exhibited a similar temperature to their uninfected control group. Surprisingly, the rise in body temperature in infected mice was directly associated with the increase in testosterone levels (**Figures 1A**, **3A, B**). Taken together, these data indicate that testosterone and the combination of testosterone + letrozole reduced hypothermia in the infected mice.

Regarding body weight, infection with *Pb* ANKA decreased body weight in untreated and letrozole-treated mice; the administration of testosterone increased body weight compared with both control groups (untreated and vehicle-treated) of infected mice. In contrast, the group treated with letrozole + testosterone and infected showed decreased body mass compared to the infected group treated only with testosterone (**Figures 3C, D**). Taken together, these data indicate that testosterone prevents weight loss during *P. berghei* infection.

Regarding glycemia, letrozole did not affect the levels of glucose in uninfected mice; in contrast, testosterone increased glucose levels in uninfected mice compared with the vehicle-treated uninfected control group. Conversely, *Pb* ANKA infection significantly increased glycemia in all groups. Remarkably, administration of letrozole, testosterone, or letrozole + testosterone in the infected groups decreased glucose levels compared to the untreated infected control group (**Figures 3E, F**). These data suggest that testosterone prevents hypothermia and cachexia caused by *Pb* ANKA infection and indicate that, in contrast to our expectations, testosterone may reduce some symptoms of malaria in a concentration-dependent manner.

Finally, we assessed the effect of testosterone on hemoglobin concentration. Contrary to our expectations, letrozole, testosterone, or the combination of letrozole + testosterone did not modify the concentration of hemoglobin in the uninfected mice compared with the vehicle-treated uninfected control group. Nevertheless, in infected groups, mice treated with testosterone decreased their hemoglobin levels compared with the groups of mice treated with vehicle or letrozole. Remarkably, the group of mice treated with letrozole + testosterone and infected exhibited a dramatic decrease in hemoglobin concentration compared to all *Pb* ANKA-infected groups (**Figures 3G, H**). Taken together, these data suggest that the reduction in hemoglobin levels in infected mice was inversely proportional to the increase in testosterone concentration.

### 3.4 The combination of letrozole + testosterone increased CD3^+^CD8^+^ and CD19^+^ populations in the spleen of CBA/Ca mice infected with *Pb* ANKA

**Figure 4:**
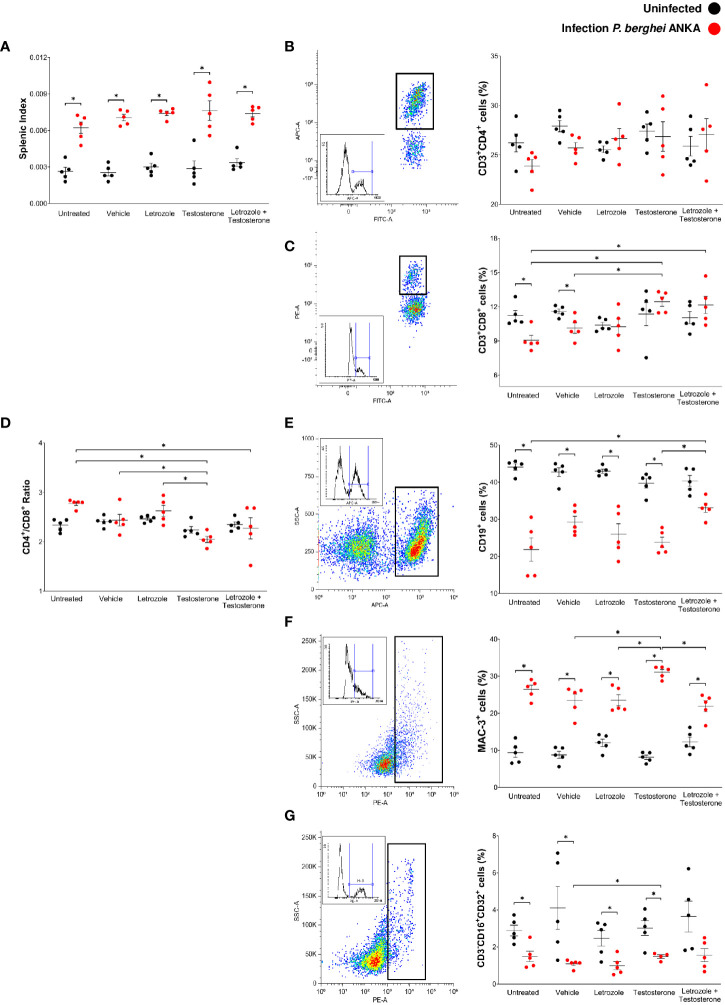
Letrozole and testosterone increased the CD3^+^CD8^+^ and CD19^+^ populations in spleens of male CBA/Ca mice infected with *Pb* ANKA. Four groups of male CBA/Ca mice (*n* = 10) were treated with letrozole, testosterone, or the combination of letrozole and testosterone, and untreated and vehicle-treated groups were used as controls. Half of the total number of mice in all groups were infected with *Pb* ANKA. Cell populations in the spleen were assessed by flow cytometry. The dot plot represents an example of how each population was selected according to the cell complexity and fluorescence emitted by the fluorochrome coupled to the specific antibody for each population. The box represents the histogram of the positive cells for each population. The dots in the cell population plots represent the individual results for each mouse ± SEM of the percentage of cells in each group. **(A)** Splenic index; **(B)** CD3^+^CD4^+^ cells; **(C)** CD3^+^CD8^+^; **(D)** the ratio CD4^+^/CD8^+^; **(E)** CD19^+^; **(F)** Mac-3^+^; **(G)** CD3^-^CD16^+^CD32^+^. Asterisks (*) represent significant differences between the two groups using a one-way ANOVA and Bonferroni *post-hoc* test with *p* ≤ 0.05 (*n* = 5). The whole experiment was performed twice.

In malaria, the spleen is the leading site of elimination of parasitized erythrocytes; in this organ, proliferation of the immune response cells and inflammation increases its size because of antigenic stimulation. Therefore, splenomegaly is an indication of the severity of infection; this condition occurs in humans and mice (Greenwood et al., 1987; Leoni et al., 2015). In this work, infection with *Pb* ANKA significantly increased the splenic index in all groups (**Figure 4A**). In addition, contrary to our expectations, administration of letrozole, testosterone, or letrozole + testosterone did not significantly modify the splenic index. However, the administration of letrozole + testosterone increased parasitemia and thus antigenic stimulation. Therefore, we assessed whether testosterone levels affected the main immune response cells (CD3^+^, CD4^+^, CD8^+^, CD19^+^, Mac-3^+^, and CD16 + /32^+^) in the spleen.

Uninfected groups treated with letrozole, testosterone, or letrozole + testosterone did not modify the immune cells assessed. Infection with *Pb* ANKA or administration of letrozole, testosterone, or letrozole + testosterone did not change the population of CD3^+^CD4^+^ cells in either group (**Figure 4B**). Interestingly, infection with *Pb* ANKA decreased the number of CD3^+^CD8^+^ cells in the untreated and vehicle-treated infected control groups. In contrast, administration of testosterone or letrozole + testosterone increased the number of CD3^+^CD8^+^ cells in infected mice compared to the untreated infected control group (**Figure 4C**). In addition, a low CD4^+^/CD8^+^ ratio has been suggested as a risk biomarker for other diseases (Wolday et al., 2020). We evaluated whether testosterone modifies this ratio. The administration of testosterone or letrozole + testosterone in the infected groups decreased the CD4^+^/CD8^+^ ratio, suggesting that CD8^+^ cells increased in these groups (**Figure 4D**).

Regarding CD19^+^ cells, infection with *Pb* ANKA decreased this population in all groups (**Figure 4E**), and treatments with letrozole or testosterone alone did not modify this population; however, infected mice treated with letrozole + testosterone increased the CD19^+^ cell population compared to the untreated infected group (**Figure 4E**). In addition, infection with *Pb* ANKA increased the population of Mac-3 compared with their respective uninfected control groups. Unexpectedly, the letrozole and letrozole + testosterone-treated groups decreased the Mac-3^+^ population compared to the infected group treated with testosterone alone (**Figure 4F**), suggesting that the decrease in the number of macrophages may be dependent on testosterone concentration. Finally, infection with *Pb* ANKA significantly decreased the CD3^-^CD16 + /32^+^ (NK cells) in mice treated with vehicle, letrozole, or testosterone. However, the group treated with testosterone and infected had an increased number of NK cells compared to the vehicle-treated infected group (**Figure 4G**). This finding suggests that the decrease in this population was dependent on testosterone concentration.

### 3.5 The combination of letrozole + testosterone increased IL-4 and TNF-α levels and decreased IL-2 and IL-17 concentrations in male CBA/Ca mice infected with *Pb* ANKA

Proinflammatory cytokines such as IFN-γ promote *Plasmodium* clearance by activating macrophages. However, overproduction of proinflammatory cytokines is also associated with the development of severe malaria, such as anemia, cerebral malaria, and death (Chao et al., 1995; Angulo and Fresno, 2002). To determine whether modifications in cell populations generated by testosterone affect cytokine synthesis, we evaluated the effect of letrozole and testosterone on the plasma levels of IL-2, IL-4, IL-6, IFN-γ, IL-10, TNF-α, and IL-17 in *Pb* ANKA-infected mice.

**Figure 5:**
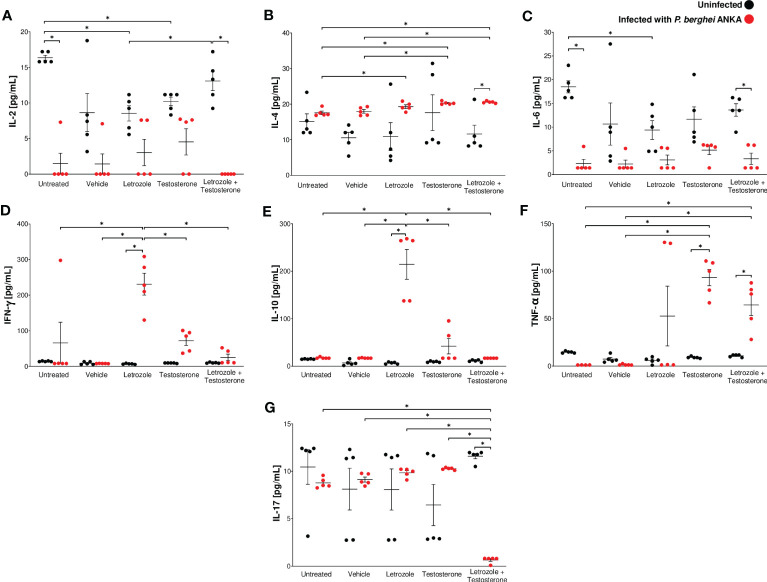
The combination of letrozole + testosterone increased IL-4 and TNF-α concentrations and decreased IL-17 concentrations in male CBA/Ca mice infected with *Pb* ANKA. Five groups of male CBA/Ca mice (*n* = 10) were treated with letrozole, testosterone, or the combination of letrozole and testosterone, and untreated and vehicle-treated groups were used as controls. Half of the mice in all groups were infected with *Pb* ANKA. All mice were sacrificed at day 8 postinfection, and plasma was obtained and quantified using flow cytometry. The levels of cytokines are as follows: **(A)** interleukin-2 (IL-2); **(B)** interleukin-4 (IL-4); **(C)** interleukin-6 (IL-6); **(D)** interferon (IFN-γ); **(E)** interleukin-10 (IL-10); **(F)** tumor necrosis factor (TNF-α); and **(G)** interleukin-17 (IL-17). The dots represent the individual results for each mouse and the horizontal line represents the mean of each cytokine for each group ± SEM (*n* = 5). Asterisks (*) represent significant differences between the two groups at *p* ≤ 0.05. Statistical analysis of one-way ANOVA and Bonferroni *post-hoc* test. The whole experiment was performed twice.

Infection with *Pb* ANKA decreased the concentration of IL-2 in all groups, particularly in the group treated with letrozole + testosterone (**Figure 5A**). In contrast, infection increased the concentration of IL-4 in the group treated with letrozole + testosterone; the increase in this group was dependent on testosterone concentration (**Figure 5B**). Regarding IL-6, infection decreased this cytokine particularly in the infected group treated with letrozole + testosterone (**Figure 5C**). In relation to IFN-γ and IL-10, infection increased the levels of both cytokines in the group treated with letrozole, and their concentration decreased inversely proportionally to testosterone levels (**Figures 5D, E**). In addition, infection increased the concentration of TNF-α in the infected groups treated with testosterone and with letrozole + testosterone (**Figure 5F**). Interestingly, a dramatic decrease in IL-17 concentration was observed only in the group treated with letrozole + testosterone and infected (**Figure 5G**).

### 3.6 The combination of letrozole + testosterone increased *Pb* ANKA-specific IgG1 antibodies

**Figure 6:**
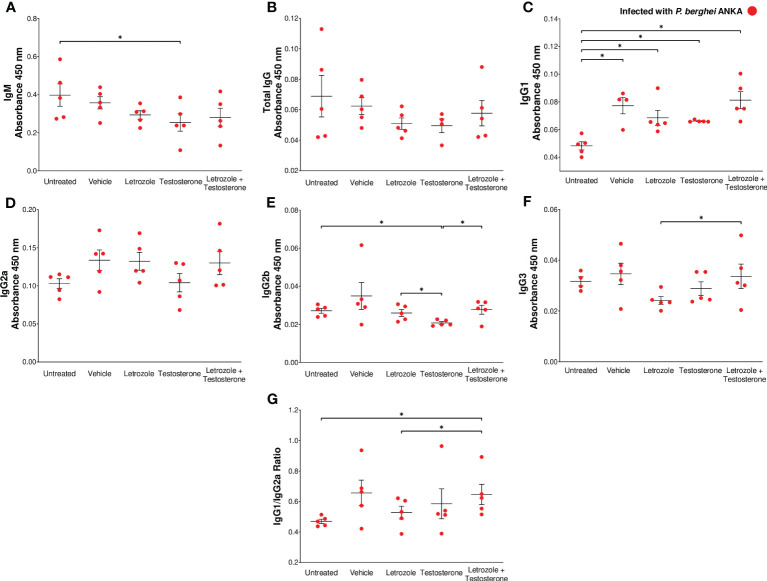
The combination of letrozole + testosterone increases *Pb* ANKA-specific IgG1 and IgG3 and the IgG1/IgG2a ratio. Five groups of male CBA/Ca mice were treated with letrozole, testosterone, or the combination of letrozole and testosterone, and untreated and vehicle-treated groups were used as controls. All mice were infected with *Pb* ANKA and sacrificed at day 8 postinfection. The dots represent the individual results for each mouse, and the horizontal line represents the mean absorbance. **(A)** IgM; **(B)** total IgG; **(C)** IgG1; **(D)** IgG2a; **(E)** IgG2b; **(F)** IgG3 from each group ± SEM (*n* = 5). The ratio of the absorbance of IgG1 to IgG2a was calculated, and **(G)** represents the mean IgG1/IgG2a ratio of each group ± SEM (*n* = 5). Asterisks (*) represent significant differences between the two groups using one-way ANOVA and Bonferroni *post-hoc* test with *p* ≤ 0.05.

In this study, the plasma concentrations of IgM and total IgG antibodies, as well as IgG1, IgG2a, IgG2b, and IgG3 subclasses were evaluated because these antibodies interfere with the parasite cycle, inhibit its infective capacity (Julien and Wardemann, 2019), and promote the elimination of *Plasmodium* by phagocytosis (Teo et al., 2016). Our results showed that testosterone administration significantly decreased IgM levels compared with the untreated infected control (**Figure 6A**). Letrozole or testosterone did not modify the level of total IgG (**Figure 6B**). However, the group treated with letrozole + testosterone had an increased concentration of IgG1 compared to the untreated infected control group; nonetheless, no differences were detected when compared to the vehicle-treated group (**Figure 6C**); neither treatment significantly changed the concentration of IgG2a (**Figure 6D**). In addition, the testosterone-treated group decreased IgG2b concentration compared to the untreated group, but when letrozole and testosterone treatments were combined, the concentration of that antibody subclass increased compared to the infected group treated only with testosterone (**Figure 6E**). In relation to IgG3 levels, we detected that the group that received letrozole + testosterone had increased levels of this antibody compared to the group that received letrozole alone (**Figure 6F**). In addition, it has been described that IFN-γ increases the synthesis of IgG2a, while IL-4 favors the synthesis of IgG1; therefore, we calculated the ratio between IgG1 and IgG2a (Snapper and Paul, 1987). Finally, the administration of letrozole + testosterone increased the IgG1/IgG2 ratio compared to the group treated with letrozole alone, indicating that testosterone induces a predominantly Th2-type response, as previously reported (Snapper and Paul, 1987) (**Figure 6G**).

## 4 Discussion

Two main strategies have been used to study the role of testosterone in the susceptibility and mortality of male patients in malaria; the first was to decrease its concentration by gonadectomy, and the second was to increase its levels by administering it exogenously. The first does not contemplate that gonadectomy reduces both androgen and estrogen concentrations, while the second does not consider that by increasing testosterone concentration, the enzyme P450 aromatase can transform it into estrogens. To avoid estrogen interference in the study of the effects of testosterone on the immune response in malaria, we inhibited the P450 aromatase enzyme *in vivo* with letrozole. We found that regardless of whether the mice were infected or uninfected, administration of letrozole + testosterone dramatically increased the concentration of free testosterone, as well as the levels of the precursor hormone DHEA. In addition, the increased free testosterone levels were associated with increased parasitemia and body temperature but decreased glucose concentration. In addition, the increase in testosterone levels induced a remarkable decrease in the hemoglobin concentration. Furthermore, testosterone selectively increased CD3^+^CD8^+^ and macrophage populations. However, the administration of letrozole + testosterone increased CD8^+^ and CD19^+^ cells. Additionally, increasing free testosterone enhanced IL-4 and TNF-α concentrations and dramatically reduced IL-17 levels. Finally, testosterone decreased IgM and IgG2b. Finally, letrozole + testosterone increased IgG1 and the IgG1/IgG2a ratio, promoting an anti-inflammatory response.

First, we corroborated that our model works because administering letrozole + testosterone increased the concentration of free testosterone; consequently, 17β-estradiol levels decreased. Interestingly, this strategy increased DHEA concentration only in mice treated with letrozole + testosterone uninfected; a likely explanation is that as estrogen concentration decreased, much of the DHEA no longer entered the steroid biosynthetic pathway because the activity of the CYP7B1 enzyme involved in steroidogenesis depends on the interaction of estrogen with its receptor (Tang et al., 2006). To assess the effects of testosterone on malaria severity, we analyzed parasitemia, hypothermia, cachexia, hyperglycemia, and anemia, all of which are associated with increased mortality in *Pb* ANKA infection (Elased et al., 2001; Onwuamaegbu et al., 2004). We found that parasitemia increased in direct proportion to the concentration of testosterone, confirming previous findings in C57Bl/10 mice infected with *P. chabaudi* and in CBA/Ca mice infected with *Pb* ANKA (Wunderlich et al., 2005; Aguilar-Castro et al., 2022). This result partially explains the immunosuppressive effect of testosterone. Another possible explanation is that the decrease in 17β-estradiol concentration increases parasitemia in *Pb* ANKA-infected mice (Cervantes-Candelas et al., 2021a).

Mice infected with *Pb* ANKA develop hypothermia (Basir et al., 2012). Our results show that in uninfected mice, the group treated with letrozole + testosterone decreased the temperature; in contrast, in infected mice, the temperature increased compared to untreated infected mice. This discrepancy reflects a complex phenomenon of temperature control mediated by different factors, including testosterone concentration and parasite infection. It is likely that the increase in TNF-α promotes the activation of cyclooxygenase-2 and the synthesis of prostaglandin E2, which acts on thermoregulatory neurons in the preoptic nucleus of the hypothalamus to induce temperature rise (Nagashima et al., 2000; Clark et al., 2006; Mosili et al., 2020). Furthermore, in this work, the increase in free testosterone levels corresponds to the increase in IL-4 concentration, which has been reported to modulate temperature by promoting catecholamine synthesis in resident macrophages in adipose tissue. Catecholamines induce the expression of uncoupling protein 1 (UCP1), which increases the temperature (Reitman, 2017; Thomas and Apovian, 2017), via fatty acid-induced thermogenesis (Matthias et al., 2000). A likely explanation for the decrease in temperature in uninfected mice treated with letrozole + testosterone is that testosterone signaling decreases UCP1 expression because testosterone, by interacting with its receptor, suppresses UCP1 expression by inhibiting cAMP response element binding protein (CREB) phosphorylation (Harada et al., 2022).

In this work, infection with *Pb* ANKA induced weight loss; when this decrease is greater than 10%, it is called cachexia. In malaria, cachexia is explained because infection with the parasite promotes the synthesis of proinflammatory cytokines that decrease lipogenesis and increase insulin resistance, which induces gluconeogenesis and proteolysis and decreases protein synthesis (Onwuamaegbu et al., 2004). In addition, *Plasmodium*-infected individuals lose body weight, leading to an imbalance in glucose metabolism. In contrast, decreased testosterone concentration in individuals with hypogonadism leads to obesity, decreased insulin sensitivity, and dyslipidemia, which is corrected by testosterone administration (Kelly and Jones, 2013; Sebo and Rodeheffer, 2021). In addition, *Pb* ANKA infection generated hyperglycemia, a condition associated with increased severity in cerebral malaria (Eltahir et al., 2010). In this work, the increase in free testosterone in infected mice decreased the glucose concentration compared to the untreated infected group; a probable explanation of this effect is that testosterone activates glucose metabolism by increasing the expression of the glucose transporter GLUT4, as well as its translocation to the plasma membrane (Sato et al., 2008). In addition, testosterone positively modulates the activity of the major glycolytic enzymes hexokinase and phosphofructokinase (Sato et al., 2008; Troncoso et al., 2021). Moreover, lower testosterone levels have been associated with insulin resistance and increased glucose concentration (Kelly and Jones, 2013; Grossmann, 2014). This condition induces inflammation through the activation of M1 macrophages (Maresch et al., 2018). In contrast, high testosterone levels reduce the synthesis of proinflammatory cytokines (D'Agostino et al., 1999) and promote the polarization of macrophages towards an anti-inflammatory M2 profile (Ren et al., 2017). Therefore, it is probable that testosterone, by regulating glucose metabolism, reduces inflammation. Consequently, it would be essential to evaluate the effect of testosterone on macrophage polarization in the future.

Anemia is one of the main causes of death from malaria (Thawani et al., 2014). In this work, we found that administration of letrozole + testosterone to infected mice decreased the hemoglobin concentration compared to untreated or vehicle-infected controls. A possible explanation for this finding is that both testosterone and infection with *Pb* ANKA inhibit the maturation and proliferation of erythroid precursors. In addition, the increased number of parasites in this group promoted erythrocyte destruction, which would at least partly explain the reduction in hemoglobin concentration as previously described (Aguilar-Castro et al., 2022). Nevertheless, we detected no differences in splenic index between infected groups even though parasitemia increased in direct proportion to the increment in testosterone levels; it is likely that testosterone reducing the inflammatory process prevented the increase in the splenic index and impaired the elimination of parasites in the spleen.

Since the spleen is the main site of *Plasmodium* elimination, immune system activation, and hematopoiesis (Engwerda et al., 2005), we analyzed the effects of testosterone on cell populations in the spleen. Interestingly, administration of testosterone or letrozole + testosterone to infected mice increased the number of CD8^+^ T cells compared to the untreated infected group. This result is consistent with that described in testosterone-treated and *Pb* ANKA-infected female mice (Aguilar-Castro et al., 2022) and in female and castrated male mice reconstituted with testosterone and infected with *P. chabaudi* (Benten et al., 1993). One possible explanation is that increasing the concentration of free testosterone enhanced the concentration of TNF-α; when this cytokine interacts with its receptor (TNRF2), it increases CD8^+^ T-cell proliferation. In addition, TNRF2-deficient mice exhibited decreased CD8^+^ T-cell proliferation (Kim and Teh, 2001). In addition, we detected a low CD4^+^/CD8^+^ ratio in both infected groups treated with testosterone indicating that CD8^+^ cells increased. This unbalanced CD4^+^/CD8^+^ T-cell ratio affects the control of parasites during *Pb* ANKA infection (Wangoo et al., 1990), which somewhat explains the increased parasitemia in these groups. Finally, CD8^+^ T cells play an important role in neuropathology and mortality in cerebral malaria (Qin et al., 2021; Liang et al., 2022). Our results suggest that testosterone is involved in the development of cerebral malaria by promoting an increase in CD3^+^CD8^+^ cells, which would at least partially explain the higher severity and lethality of malaria in male patients.

On the other hand, B cells are required to clear *Plasmodium* infection (Langhorne et al., 1998), and treatment with letrozole + testosterone increased the CD19^+^ cell population in the spleen, which is noteworthy because B cells do not possess androgen receptors (Benten et al., 2002). Nevertheless, indirectly, it is likely that the increase in B cells is explained by the increase in IL-4 levels because of the increase in free testosterone concentration, since IL-4 promotes B-cell proliferation (Troye-Blomberg et al., 1990). Moreover, infection with *Pb* ANKA increased the number of Mac-3^+^ cells in all groups, but this increase was significantly higher in testosterone-treated mice. This finding could be explained by the fact that testosterone increases the expression of macrophage chemotactic protein-1 (MCP-1) (Su et al., 2015), a key chemokine in macrophage migration and recruitment. Remarkably, the group treated with letrozole + testosterone had a decreased Mac-3^+^ population relative to the group infected treated with testosterone alone; macrophages control *Plasmodium* replication and protect against anemia (Couper et al., 2007). Therefore, it is likely that the decrease in the Mac-3^+^ population is associated with the increased parasitemia and severe anemia in this group.

Finally, we detected that infection decreased the NK cell population, independent of testosterone concentration. However, testosterone may reduce NK cell activity as previously reported (Hou and Zheng, 1988) because NK cells produce IFN-γ in mice infected with *Plasmodium* (Mohan et al., 1997), and this cytokine promotes parasite elimination via phagocytosis (Roetynck et al., 2006), which would at least partially explain the reduction in IFN-γ in the group that received letrozole + testosterone as well as the increase in parasitemia and anemia in the same group. Therefore, it is worth studying the effect of testosterone on NK cell function in malaria.

In addition, our results showed that administration of letrozole + testosterone to infected mice dramatically reduced IL-17 concentrations. This result corroborates the finding of Schwinge et al., who showed that testosterone reduced the IL-17 concentration associated with decreased RORc mRNA expression, which suppresses Th17 cell differentiation (Schwinge et al., 2015). In addition, decreased IL-17 concentrations have also been associated with severe anemia (Xu et al., 2013), which would explain the anemia that occurred in the letrozole + testosterone-treated group. Furthermore, macrophage-derived IL-17 induces the expression of CCL2/7, which recruits macrophages, and promotes *P. berghei* NK65 elimination (Ishida et al., 2013). Taken together, these findings suggest that the decrease in IL-17 is related to a decrease in the macrophage population, which increases parasitemia and anemia. On the other hand, the administration of letrozole + testosterone to infected mice increased TNF-α compared to the infected control group, probably because the rise in testosterone levels increased the concentration of TNF-α as described previously (Patil et al., 2016).

The infected group treated with letrozole exhibited increased concentrations of IFN-γ, TNF-α, and IL-10. These results are probably a consequence of letrozole increasing the levels of these cytokines (Wang et al., 2009; Mehta et al., 2012). Additionally, the increase in IL-10 is possibly due to a mechanism of inflammation regulation; it has been shown that Th1 CD4^+^ IFN^+^ T cells produce IL-10 as a protective mechanism against immunopathology during *Plasmodium* infection (Freitas do Rosario et al., 2012).

Finally, the clearance of *Plasmodium* requires antibodies (von der Weid et al., 1996). Here, we show that testosterone decreased IgM and IgG2b levels, and this finding was consistent with the reduction in IgG2b observed in testosterone-treated mice infected with *P. chabaudi* (Benten et al., 1997). Notably, the group treated with letrozole + testosterone exhibited increased IgG1 and IgG3 levels compared to untreated infected mice and the infected testosterone-treated group, respectively. This differs from an *in vitro* study showing that testosterone decreases IgG1 production (Kanda et al., 1996). A possible explanation for this discrepancy is the different *Plasmodium* strains used and that the assay was performed *in vitro*. In addition, elevated levels of IgG3 and IgG1 have been associated with decreased clinical symptoms (Stanisic et al., 2009) while increased IgG3 is associated with increased efficiency in phagocytosis. It is therefore likely that the decrease in the number of macrophages is the cause of the high levels of parasitemia as reported previously (Bredius et al., 1994).

We are aware that increasing the concentration of testosterone also increased the concentration of DHEA, which, due to its immunomodulatory properties (Buendia-Gonzalez and Legorreta-Herrera, 2022), could influence our results. However, we did not detect such an increase in the infected mice.

**Figure 7:**
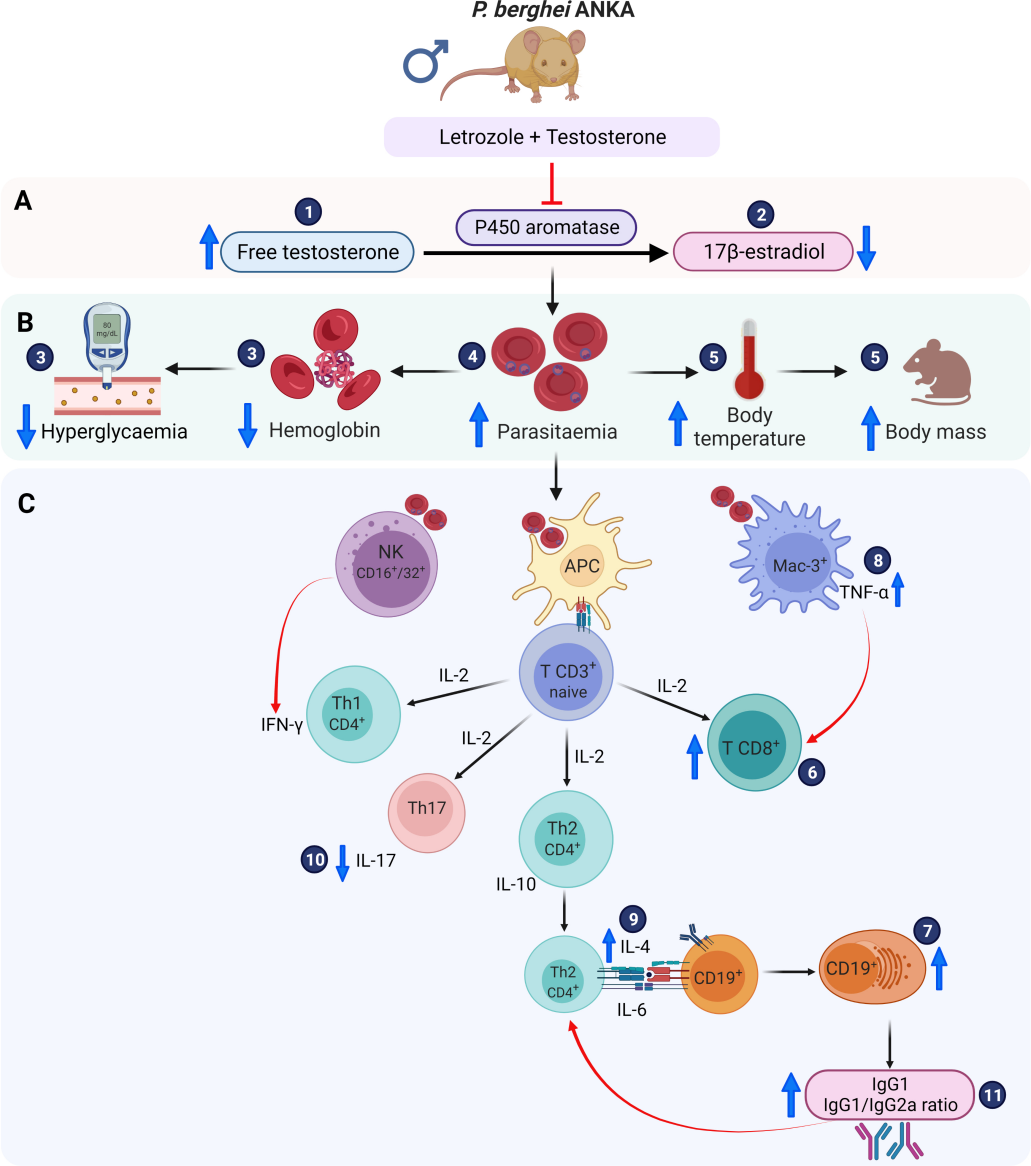
Immunomodulatory effect of letrozole + testosterone on infection with *Pb* ANKA. **(A)** Testosterone and letrozole administration increased free testosterone concentration (1) and decreased 17β-estradiol levels (2). **(B)** Measurement of the severity of infection. The increase in testosterone concentration reduced glycemia and blood hemoglobin concentration (3), which was associated with increased parasitemia (4); in addition, the increase in testosterone concentration resulted in an elevation of both body temperature and body mass (5). **(C)** All these variables are associated with important effects on the immune response. The administration of letrozole + testosterone increased both populations: CD8^+^ (6) and CD19^+^ cells (7), which increased TNF-α (8) and IL-4 synthesis (9). Additionally, the combination of letrozole + testosterone decreased IL-17 levels (10), which was associated with a drop in hemoglobin concentration. Finally, the administration of letrozole and testosterone augmented IgG1 levels and the IgG1/IgG2a ratio (11), promoting a Th2-type response. Created with BioRender.com.

Based on our findings and previously reported results of the immune response generated by *Pb* ANKA infection, we propose a schematic model to explain the effect of the combination of letrozole + testosterone on the immune response (**Figure 7**). The administration of letrozole + testosterone to *Pb* ANKA-infected mice induced elevated levels of free testosterone, which influenced an increase in CD3^+^CD8^+^ and CD19^+^ cells. Additionally, it reduced the concentration of IL-17 but increased IL-4 and TNF-α. Finally, it increased IgG1 levels and the IgG1/IgG2a ratio, which promotes a Th2-type response. These immune responses reduce hypothermia and hyperglycemia but promote parasitemia and severe anemia.

## 5 Conclusion

This work highlights the immunomodulatory role of testosterone in malaria. It is noteworthy that increasing the concentration of free testosterone regulates glucose concentration, reduces hypothermia, and reduces the inflammatory process in the spleen, probably because of the reduction in IFN-γ concentration. Furthermore, it increases the number of CD8^+^ and CD19^+^ cells, decreases the number of macrophages, and probably modifies the activity of NK cells; these cells regulate the immune response by increasing the concentration of cytokines that induce B-cell activation. Additionally, the increase in free testosterone decreased the concentration of IL-17 but increased the levels of IL-4 and TNF-α. This impacted IgG1 levels and the IgG1/IgG2a ratio, which promoted a Th2-type anti-inflammatory response that reduced hypothermia and hyperglycemia but increased parasitemia and induced severe anemia.

## Data availability statement

The original contributions presented in the study are included in the article/**Supplementary Material**. Further inquiries can be directed to the corresponding authors.

## Ethics statement

The protocol received approbation by the Local Ethics Committee (registration number 28/04/SO/3.4.1) according to the Mexican official standard NOM-94 062-ZOO-1999 for the use and care of laboratory animals.

## Author contributions

All the authors significantly contributed to this work. M-LH designed the study, acquired funding, supervised experiments, analyzed the results, and wrote and edited the manuscript. TN-P contributed to formal analysis, data acquisition, discussion of results, and preparation of figures and draft of the manuscript. VS-C performed data acquisition, analysis, and discussion of the results and wrote the manuscript. LC-C, FB-G, JA-C, and OF-R contributed to data acquisition, analysis, and discussion of results and preparation of figures. All authors revised the manuscript critically for important intellectual content. All authors contributed to the article and approved the submitted version.

## Supplementary material

Supplementary Figure 1 Effect of different concentrations of letrozole on the induction of parasitaemia. **(A)** Parasitaemia kinetics in groups of CBA/Ca mice treated with letrozole at concentrations of: 0, 1, 2, 4 and 7 mg/kg and infected with *Pb* ANKA. **(B)** Parasitaemia induction was calculated as the percentage difference of the geometric mean of the letrozole-treated group minus the geometric mean of the vehicle-treated group at day 8 postinfection for each mouse. Data were analyzed with one-way ANOVA with Bonferroni *post hoc* test. n=4, asterisks represent significant differences between two groups. * p<0.05; **** p<0.0001.

Supplementary Figure 2 Effect of administering letrozole, testosterone or the combination of letrozole and testosterone on free testosterone concentration. The testosterone concentration was quantified by immunoassay, and the graph plots the values for each mouse in the controls (untreated and vehicle-treated group), letrozole (7mg/kg), testosterone (30 mg/kg), and the combination of both at the same dose (n = 5). The average testosterone concentration in each group is denoted at the top.

Supplementary Figure 3 Synergistic effect of the combination of letrozole and testosterone administration on free testosterone concentration. The straight lines represent the potency of letrozole (red), testosterone (black) or letrozole and testosterone (blue) administration on the mean plasma-free testosterone concentration. The logarithm of the minimum dose (1 mg/kg) to the maximum dose of letrozole (7 mg/kg) with the fixed dose of testosterone (30 mg/kg) or their combination was calculated.
