# Supplementary figures and images for: Immunomodulatory effects of testosterone and letrozole during Plasmodium berghei ANKA infection
Source: Front Cell Infect Microbiol. 2023 Jun 13;13:1146356. doi: 10.3389/fcimb.2023.1146356 (PMC10296187; doi:10.3389/fcimb.2023.1146356)

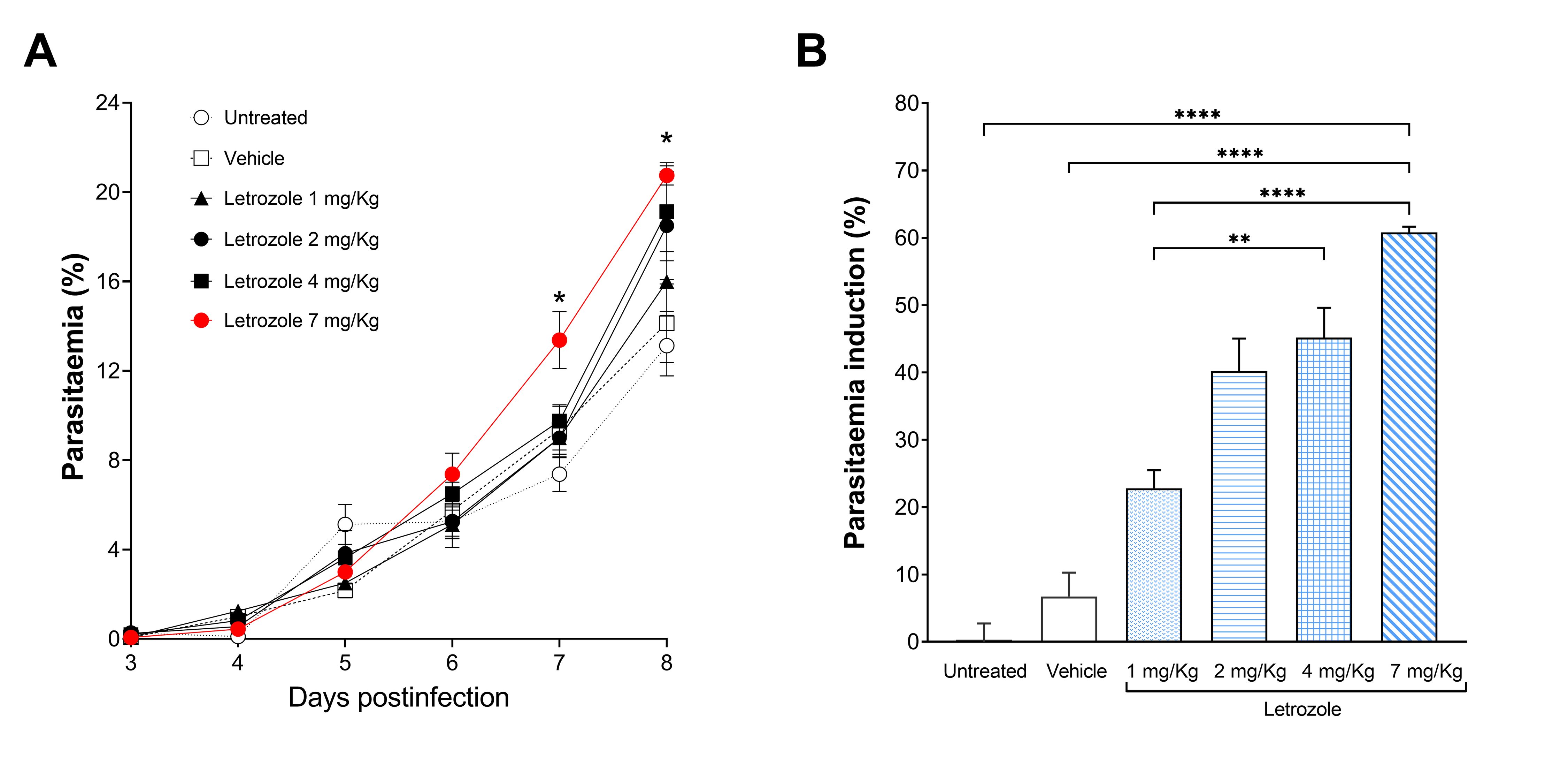

Supplement: Supplementary Figure 1 — Effect of different concentrations of letrozole on the induction of parasitaemia. (A) Parasitaemia kinetics in groups of CBA/Ca mice treated with letrozole at concentrations of: 0, 1, 2, 4 and 7 mg/kg and infected with Pb ANKA. (B) Parasitaemia induction was calculated as the percentage difference of the geometric mean of the letrozole-treated group minus the geometric mean of the vehicle-treated group at day 8 postinfection for each mouse. Data were analyzed with one-way ANOVA with Bonferroni post hoc test. n=4, asterisks represent significant differences between two groups. * p<0.05; **** p<0.0001. [file Image_1.tif]

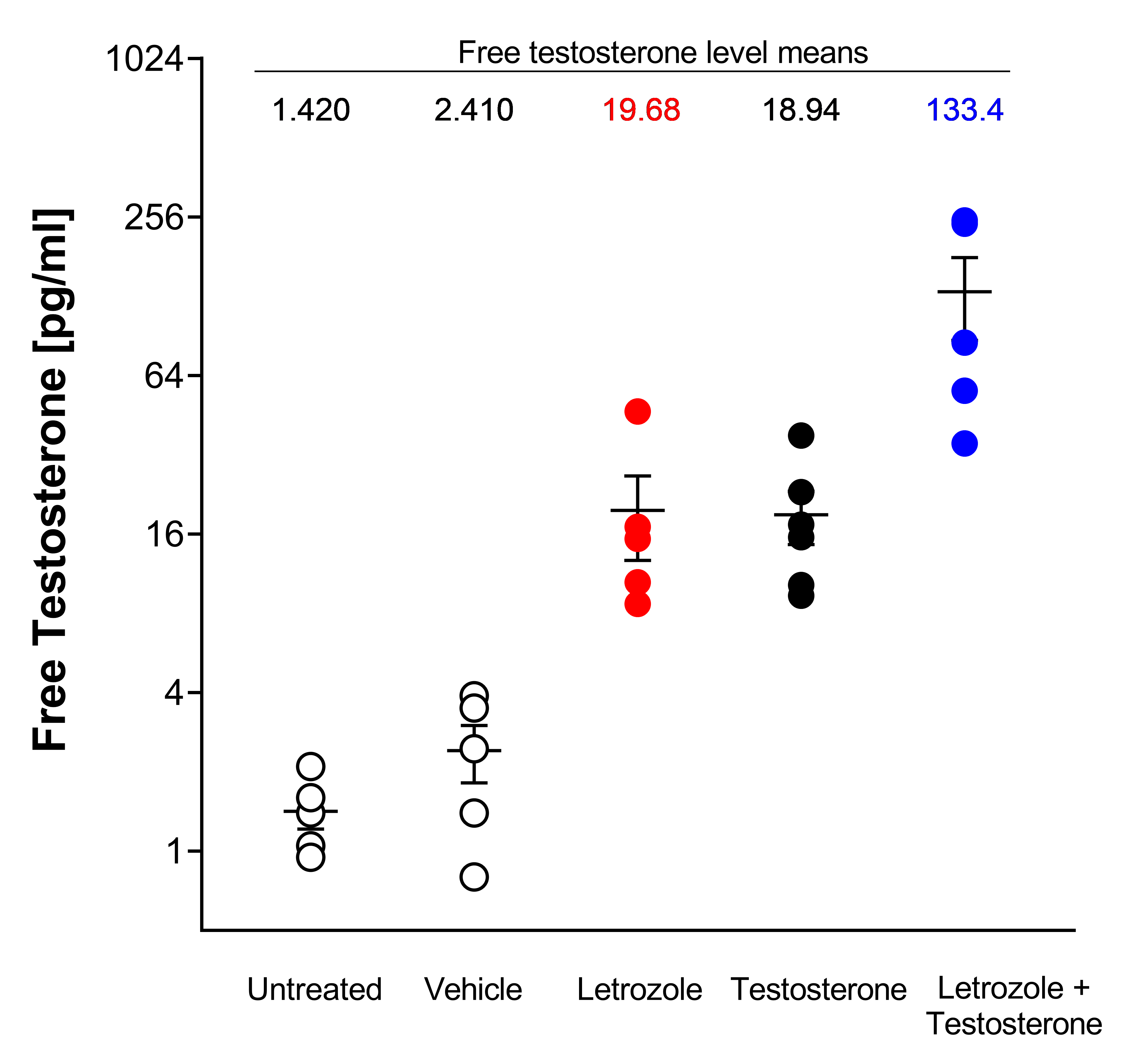

Supplement: Supplementary Figure 2 — Effect of administering letrozole, testosterone or the combination of letrozole and testosterone on free testosterone concentration. The testosterone concentration was quantified by immunoassay, and the graph plots the values for each mouse in the controls (untreated and vehicle-treated group), letrozole (7mg/kg), testosterone (30 mg/kg), and the combination of both at the same dose (n = 5). The average testosterone concentration in each group is denoted at the top. [file Image_2.tif]

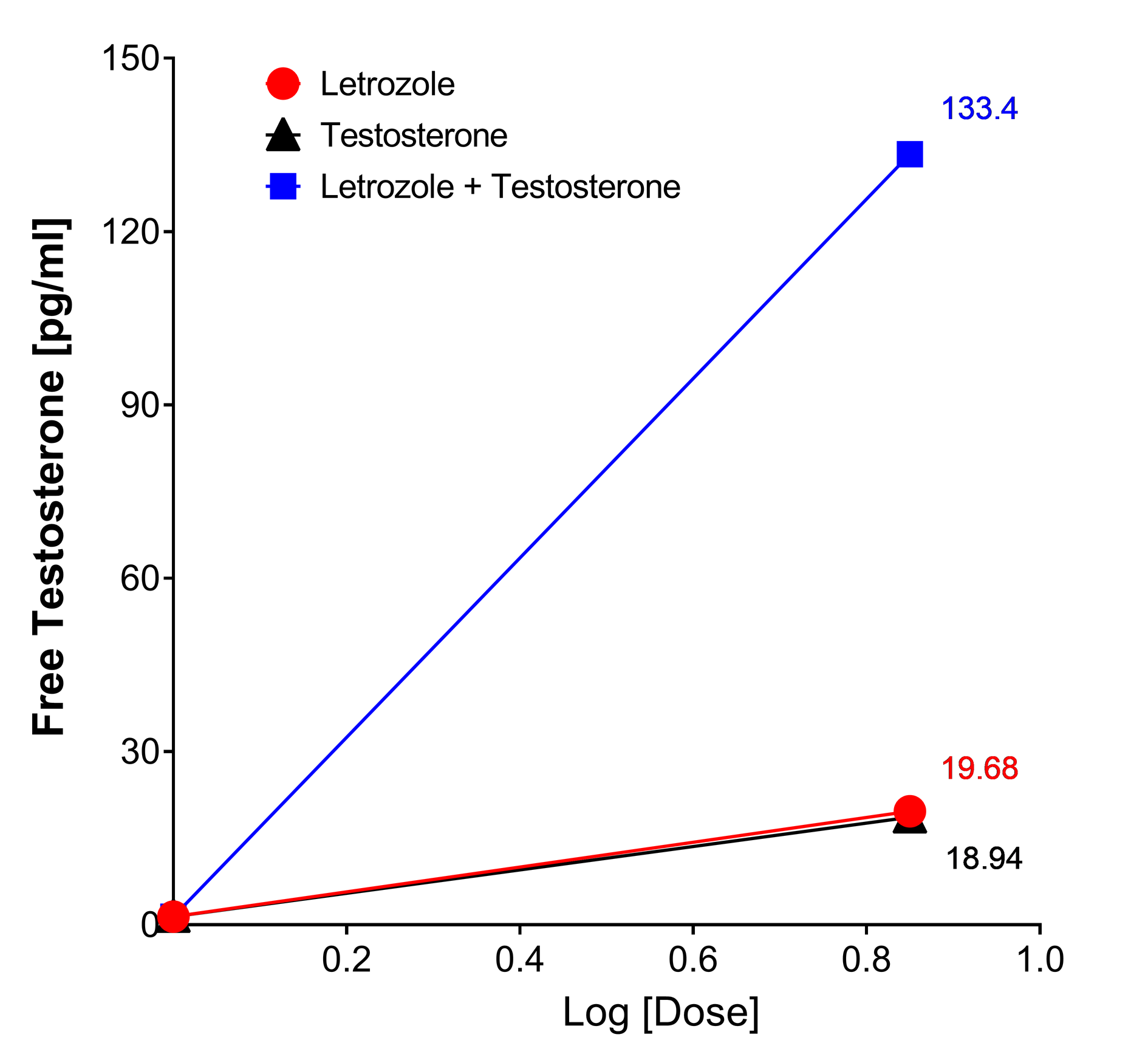

Supplement: Supplementary Figure 3 — Synergistic effect of the combination of letrozole and testosterone administration on free testosterone concentration. The straight lines represent the potency of letrozole (red), testosterone (black) or letrozole and testosterone (blue) administration on the mean plasma-free testosterone concentration. The logarithm of the minimum dose (1 mg/kg) to the maximum dose of letrozole (7 mg/kg) with the fixed dose of testosterone (30 mg/kg) or their combination was calculated. [file Image_3.tif]
